# IDH1 mutation promotes lung cancer cell proliferation through methylation of Fibulin-5

**DOI:** 10.1098/rsob.180086

**Published:** 2018-10-10

**Authors:** Bingdi Yan, Yanbing Hu, Tiangang Ma, Yanjun Wang

**Affiliations:** 1 Department of Pneumology, The 2nd Hospital Affiliated to Jilin University, Changchun, China; 2 Department of Ultrasound, The 2nd Hospital Affiliated to Jilin University, Changchun, China; 3 Nursing Department, The 2nd Hospital Affiliated to Jilin University, Changchun, China

**Keywords:** IDH1, methylation, Fibulin-5, lung cancer

## Abstract

Mutation in isocitrate dehydrogenase (*IDH*) leads to an aberrant function of the enzyme, leading to the production of hydroxyglutarate, as well as changes in cellular metabolism, DNA methylation and histone modification. Previous studies uncovered mutations in *IDH1* in several malignancies, with the most frequent mutation being IDH1 R132H. It has been demonstrated that IDH1 expression is induced in non-small-cell lung cancer (NSCLC). However, the contribution of IDH1 mutation in the malignant transformation and development of NSCLC is unclear. In our study, we show that IDH1 R132H enhanced the migration and proliferation of NSCLC cells. Moreover, IDH1 R132H was a crucial modulator of 2-hydroxyglutarate, whose production from cells with IDH1 mutation promoted the binding of DNA-methyltransferase 1 (DNMT1) to the Fibulin-5 promoter, leading to its methylation. As a result, Fibulin-5 silencing in cells with IDH1 mutation enhanced the migration and proliferation of NSCLC cells. We show that the IDH1 mutation was present in tissues sampled from patients with NSCLC, which was reversely linked to Fibulin-5 expression. In this study, we suggest an innovative model for IDH1 R132H/Fibulin-5 pathway, which could throw light upon the activity of IDH1 R132H in NSCLC.

## 1. Background

Metabolic dysregulation is prevalent in malignant cells. Isocitrate dehydrogenase 1 and 2 (IDH1 and IDH2), enzymes that rely on NADP^+^, act at the crossroads of cellular metabolism in oxidative respiration and oxygen-sensing signal transduction [1]. IDH1 acts in the redox reaction that transforms isocitrate into α-ketoglutarate by reducing NADP to NADPH and producing CO_2_ [2]. NADPH is crucial in the maintenance of glutathione levels, an important antioxidant, and the reduced status of thioredoxin [3], both of which counteract oxidative injury and enhance cell viability and growth of mammalian cells [4]. The additional evaluation of IDH1 expression in non-small-cell lung cancer (NSCLC) through the screening of a large population identified IDH1 as a diagnostic biomarker, owing to its favourable sensitivity and specificity [4,5]. Nevertheless, our understanding of the specific biological influence of IDH1 mutation on NSCLC remains insufficient, as the role of IDH1 mutations in NSCLC is still under debate [6]. A previous study has uncovered IDH mutations in various malignancies that alter histone modification, cellular metabolism and DNA methylation [7]. These mutations lead to an aberrant IDH1 function that transforms α-ketoglutaric acid (α-KG) into 2-hydroxyglutarate (2-HG) [8,9]. As 2-HG is a structural analogue of α-KG, it competitively inhibits the dioxygenases that rely on α-KG, including methylcytosine dioxygenase TET2 and histone demethylases [9,10], inducing hypermethylation of DNA and histones in cells with IDH1 mutations. These changes in gene expression induced by IDH1 mutation disturb cell differentiation and enhance malignant cell generation, proliferation and invasion, as well as angiogenesis [10–12]. Numerous studies have investigated DNA hypermethylation in promoter-related CpG islands of tumour suppressors, as well as in DNA repair genes, which leads to the transcriptional silencing of these genes in human cancer [13]. However, how genes are modulated through 2-HG-triggered DNA methylation, as well as the biological activities involved in this modulation, remain unclear.

Fibulins are glycoproteins belonging to the family of extracellular matrix proteins [14,15] that are characterized by a shared globular fibulin-like domain at the C-terminus [15]. These proteins participate in the aggregation and stabilization of supramolecular extracellular matrix complexes, which in turn modulate crucial cellular reactions such as cell adhesion, proliferation and migration, contributing to the generation of malignancies, vessels, as well as fibrosis. Previous studies have revealed that the fibulins commonly silenced through promoter hypermethylation in NSCLC are Fibulin-2, Fibulin-3 and Fibulin-5 [16–18]. Fibulin-3 and Fibulin-5 inhibit the invasion and metastasis of lung cancer (LC) cells by downregulating the expression of matrix metalloproteinase 7 (MMP-7) [16,19], which promotes metastasis through degradation of the basement membrane that acts as barrier to the adjacent tissues [20]. However, the mechanism underlying the epigenetic modulation of fibulin expression remains unclear.

Our study reveals that Fibulin-5 silencing in NSCLC is due to the hypermethylation of its promoter through 2-HG, production of which arises from a mutation in *IDH1*. Throwing light on the reversible modulation by IDH1 mutation of Fibulin-5 in enhancing proliferation and migration of NSCLC cells offers a promising strategy for the treatment of metastatic LC.

## 2. Methods

### 2.1. Western blot

Western blot (WB) was carried out as previously described [19]. SDS-PAGE gels (12%) were used to isolate cell lysates, which were subsequently blotted on nitrocellulose membranes and probed with primary antibodies. Primary antibodies against IDH1 (Cell Signaling Technology, Danvers, MA, USA), DNA-methyltransferase 1 (DNMT1; Abcam, Shanghai, China), IDH1 R132H (Dianova, Hamburg, Germany), TET2, Fibulin-5 (Abcam) and actin (Santa Cruz Technology, Dallas, TX, USA) were used in this study. Procedures were carried out independently in triplicate.

### 2.2. Cell culture and reagents

Human LC cell lines H1299 and H460 were acquired from Cell Repository, Chinese Academy of Sciences (Shanghai, China). DMEM supplemented with 10% FBS (Gibco, Carlsbad, CA, USA), 100 mg l^−1^ streptomycin and 1 × 10^5^ U l^−1^ penicillin (Gibco) was used to cultivate cells at 37°C in 5% CO_2_. Cross contamination of cells was checked by short tandem repeat DNA profiling at six months, and cultures were examined for mycoplasma contamination. Disulfiram (DSF), 2-HG, AGI-5198 (AGI) and 5-aza-2-deoxycytidine (5-aza) were purchased from Cayman Chemical.

### 2.3. Cell viability assay

H460 and H1299 cells were inoculated (5 × 10^3^ cells well^−1^) in 96-well plates. The MTS assay was used to evaluate cell proliferation after 24 h (Promega, Madison, WI, USA).

### 2.4. Transwell assay

Transwell assay was carried out in chemotaxis chambers containing 24 wells. A total of 5 × 10^4^ H460 or H1299 cells were inoculated into the top chamber in DMEM (200 µl) without serum. Bottom chambers contained DMEM (600 µl) supplemented with 10% FBS. Cells that migrated through the pores to the bottom chamber were fixed with paraformaldehyde (4%) and stained with crystal violet. Cells were counted using a microscope (Leica, DM4000B).

### 2.5. Plasmid and siRNA transfection

Wild-type (WT) and mutant IDH1 cDNA were subcloned into the GV358 vector (Genechem, Shanghai, China). Vectors encoding mutant IDH1 R132H (LV-GFP-IDH1R132H), WT IDH1 (LV-GFP-IDH1WT) or a control empty vector (LV-GFP-VECTOR) were obtained from Genechem. Puromycin (2 mg ml^−1^) was used to select steadily expressing cell lines. Transfection was carried out with Lipofectamine 2000 (Invitrogen). Human IDH1*,* DNMT1, TET2 and scrambled control siRNAs were purchased from Santa Cruz Biotechnology.

### 2.6. DNA methylation assay

Isolation of genomic DNA and bisulphite modification were carried out as previously described [17]. The unmethylated Fibulin-5 promoter was amplified using the primer pair 5′-TGTAGTGGTTGGGAGGATTTTGGTG-3′ and 5′-TTCCTAACATATCCAAAACACACAA-3′.

### 2.7. Chromatin immunoprecipitation

Chromatin immunoprecipitation (ChIP) was carried out using ChIP Assay Kit (Millipore, MA, USA) with the manufacturer's instructions slightly modified. The solutions used were from the ChIP Assay Kit unless otherwise specified. Anti-DNMT1 antibodies were subsequently added and incubated overnight at 4°C in a shaking incubator. Normal rabbit IgG acquired from Invitrogen served as negative control. Precipitates were evaluated by PCR for Fibulin-5 with the following primers 5′-GCTAAGCAAAACCAGGTGCT-3′ and 5′-GTGCGAAGGCGAGAAGAAA-3′ [21].

### 2.8. Real-time reverse transcription-PCR (qPCR)

RNA was isolated using Tri-Reagent. Briefly, the quantity and purity of RNA were assessed by spectrophotometry. RNA (3 mg) from LC cells after supplementation with deguelin was used in every RT reaction. qPCR was carried out on a C1000 Thermal Cycler CFX96 Real-time PCR Detection System (Bio-Rad).

### 2.9. Patient samples

Tissues were sampled from 40 patients with NSCLC by radical prostatectomy performed at the 2nd Hospital Affiliated to Jilin University. No patients underwent radiation or androgen deprivation treatment prior to the operation. Diagnosis was verified by three independent pathologists.

### 2.10. Statistical analysis

GraphPad Prism V software was used for statistical analysis. Every procedure was carried out no less than 3 times independently. Data are represented as the mean ± s.e.m. Differences between groups were assessment by Student's paired *t*-test. The difference was regarded as significant when *p < 0.05, **p < 0.01 and ***p < 0.001.

## 3. Results

### 3.1. IDH1 R132H mutation promotes proliferation and migration of non-small-cell lung cancer cells

**Figure 1.**
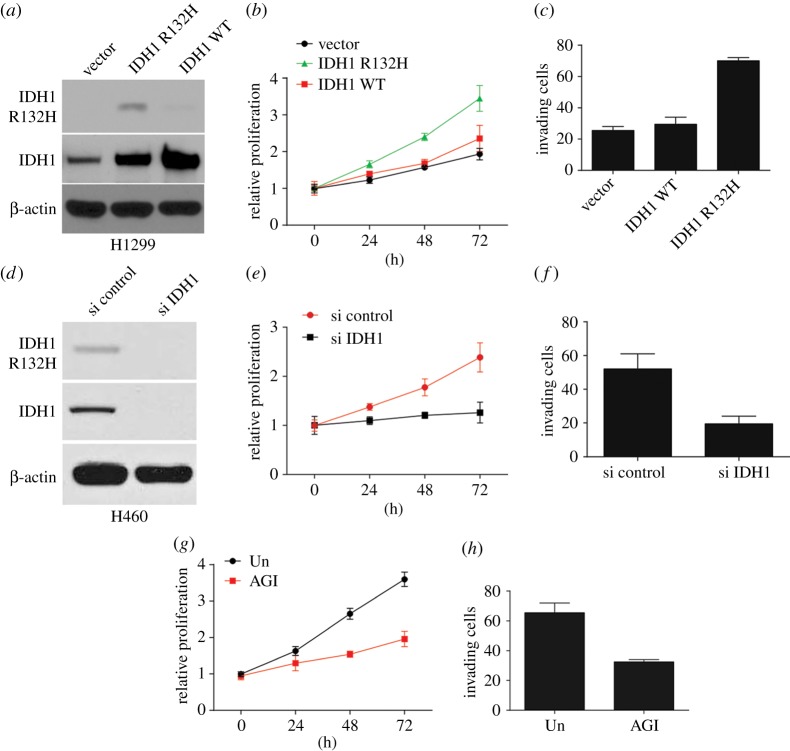
IDH1 R132H mutation induces proliferation and migration of NSCLC cells. (*a*) WB analysis of IDH1 and IDH1 R132H protein levels in H1299 cells transduced with an empty vector, IDH1 WT or mutant IDH1 R132H. (*b*) Proliferation of H1299 cells transduced with an empty vector, IDH1 WT or mutant IDH1 R132H assessed by using the MTS assay. (*c*) Migration of H1299 cells transduced with an empty vector, IDH1 WT or mutant IDH1 R132H was assessed by Transwell migration assay. (*d*) WB of IDH1 protein in H460 cells after transfection with IDH1 siRNA. (*e*) Proliferation of H460 cells after transfection with IDH1 siRNA was assessed by MTS assay. (*f*) Migration of H460 cells after transfection with IDH1 siRNA was assessed by Transwell migration assay. (*g*) Proliferation of H1299 cells expressing mutant IDH1 R132H and treated with 20 µM IDH1 inhibitor (AGI-5198) was assessed by MTS assay. (*h*) Migration of H1299 cells treated as in (*g*) was assessed by Transwell migration assay.

To determine the role of IDH1 R132H in NSCLC, IDH1 WT H1299 cells were steadily transfected with lentiviral vectors encoding mutant IDH1 (IDH1 R132H), WT IDH1 or empty vector. WB was carried out to detect the expression of IDH1 R132H (figure 1*a*). The growth rate of H1299 transfected with IDH1WT and control vector was gradually sluggish, but H1299 cells expressing IDH1 R132H had significant growth advantage (figure 1*b*). Furthermore, Transwell assays showed that IDH1 R132H increased H1299 migration (figure 1*c*). By contrast, IDH1 siRNA in H460 cells expressing the mutant IDH1 (R132H) protein decreased proliferation and migration (figure 1*d–f*). A specific inhibitor of mutant IDH1 R132H, AGI-5198 impairs the production of D-2-HG, inhibits cell proliferation and enhances the differentiation of glioma [22]. In our study, we subsequently examined whether AGI-5198 similarly inhibits the oncogenic transformation of H1299 cells that expressed the IDH1 mutation. H1299 cells with IDH1 R132H mutation were treated with 20 µM of AGI-5198 for 72 h; subsequently, cell proliferation and migration were evaluated. AGI-5198 inhibited migration and proliferation of H1299 cells that expressed IDH1 R132H (figure 1*g*,*h*). Consequently, we suggest that IDH1 mutation at R132H promotes migration and proliferation of NSCLC cells.

### 3.2. IDH1 R132H mutation enhances lung cancer progression through 2-hydroxyglutarate secretion

**Figure 2.**
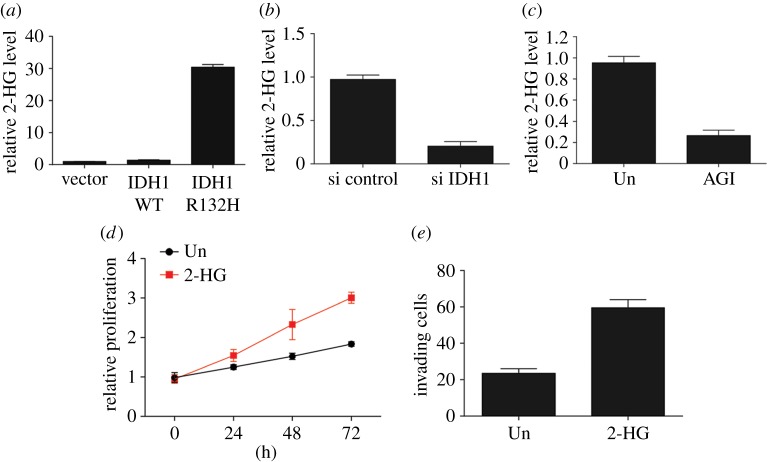
IDH1 mutation promotes 2-HG production in NSCLC cells. (*a*) 2-HG levels in H1299 cells steadily expressing an empty vector, IDH1 WT or mutant IDH1 R132H. (*b*) 2-HG levels in H460 cells after transfection with IDH1 siRNA. (*c*) 2-HG levels in H1299 cells expressing IDH1 R132H after treatment with 20 µM AGI-5198. (*d*) Proliferation of H1299 cells supplemented with 2 mM 2-HG was assessed by MTS assay. (*e*) Migration of H1299 cells supplemented with 2 mM 2-HG was assessed by Transwell migration assay.

We subsequently determined 2-HG levels in LC cells because malignancy related IDH1 mutations result in a neo-enzymatic activity that catalyses the reduction of α-ketoglutarate into 2-HG, which relies on NADPH^2^. We discovered that WT IDH1 overexpression in H1299 cells failed to noticeably elevate 2-HG secretion (figure 2*a*). However, transfection of IDH1 R132H mutant into H1299 cells remarkably promoted 2-HG production (figure 2*a*). In contrast, knockdown of IDH1 in H460 or inhibition of IDH1 in H1299 transfected with IDH1 R132H plasmid suppressed the secretion of 2-HG (figure 2*b*,*c*). Moreover, treatment with 2-HG of IDH1 WT cells (H1299 cells) increased proliferation and migration (figure 2*d*,*e*). Consequently, these findings indicate that IDH1 mutation increases the proliferation and migration of NSCLC cells through 2-HG secretion.

### 3.3. IDH1 mutation induces hypermethylation of Fibulin-5 promoter

**Figure 3.**
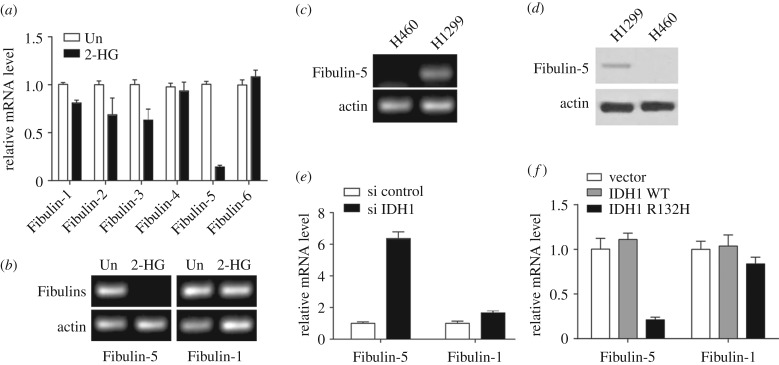
IDH1 mutation induces methylation of Fibulin-5 promoter. (*a*) mRNA levels of fibulin proteins in H1299 cells treated with 2 mM 2-HG. (*b*) Genomic PCR of unmethylated promoters of Fibulin-1 and Fibulin-5 genes in H1299 cells treated with 2 mM 2-HG. (*c*) Genomic PCR of unmethylated promoters of Fibulin-5 gene in H460 and H1299 cells. (*d*) WB analysis of Fibulin-5 in H1299 and H460 cells. (*e*) Fibulin-1 and Fibulin-5 mRNA levels in H460 cells transduced with IDH1 siRNA. (*f*) Fibulin-1 and Fibulin-5 mRNA levels in H1299 cells expressing an empty vector, IDH1 WT or mutant IDH1 R132H.

Previous studies showed that 2-HG represses gene expression through the hypermethylation of gene promoters [23,24]. It was also demonstrated previously that fibulin family proteins are closely linked with proliferation and migration of NSCLC cells. We consequently examined whether Fibulin expression levels were affected by the production of 2-HG. We investigated the expression of the six fibulin proteins in H1299 cells after treatment with 2-HG, and we discovered that Fibulin-5 expression only was noticeably repressed transcriptionally by 2-HG supplementation (figure 3*a*). Treatment with 2-HG of H1299 cells decreased the levels of unmethylated Fibulin-5 promoter. However, the levels of unmethylated Fibulin-1 promoter were not altered (figure 3*b*). Methylation of the Fibulin-5 promoter was higher in H460 cells than in H1299 cells (figure 3*c*). We also show that protein levels of Fibulin-5 were higher in H1299 cells than in H460 cells, in agreement with previous studies [16,17] (figure 3*d*). IDH1 silencing in H460 cells induced the derepression of Fibulin-5 expression (figure 3*e*) but failed to affect Fibulin-1 expression. However, expression of mutant IDH1 instead of WT IDH1 repressed Fibulin-5 expression, but had no effect on the Fibulin-1 mRNA level (figure 3*f*). Our findings indicate that mutation in *IDH1* induces Fibulin-5 repression through the hypermethylation of its promoter.

### 3.4. IDH1 mutation promotes DNMT1 binding to Fibulin-5 promoter

**Figure 4.**
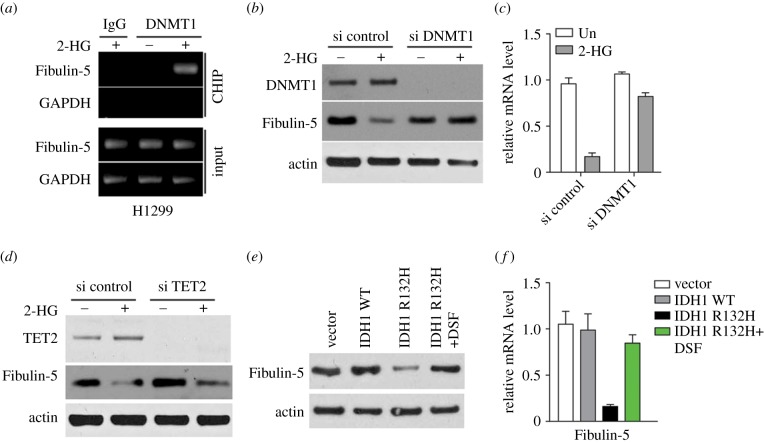
IDH1 mutation promotes binding of DNMT1 to Fibulin-5 promoter. (*a*) Binding of DNMT1 to Fibulin-5 promoter in H1299 cells treated with 2 mM 2-HG was evaluated by CHIP assay. GAPDH served as negative control. (*b*) WB analysis of DNMT1 and Fibulin-5 in H1299 cells transfected with control or DNMT1 siRNA and treated with 2 mM 2-HG. (*c*) RT-PCR analysis of Fibulin-5 expression in H1299 cells treated as in (*b*). (*d*) WB analysis of TET2 and Fibulin-5 in H1299 cells transfected with control or TET2 siRNA and treated with 2 mM 2-HG. (*e*) WB analysis of Fibulin-5 expression in H1299 cells expressing an empty vector, WT IDH1 or mutant IDH1 R132H and treated with a DNMT1 inhibitor, disulfiram (DSF, 200 nM). (*f*) Fibulin-5 mRNA levels in H1299 cells treated as in (*e*).

A previous study has demonstrated that DNMT1 is necessary for downregulation of target genes modulated by 2-HG [25]. We subsequently explored the influence of DNMT1 on Fibulin-5 expression. 2-HG supplementation of H1299 cells induced DNMT1 aggregation on the Fibulin-5 promoter (figure 4*a*). DNMT1 silencing by siRNA abrogated the effect of 2-HG on Fibulin-5 expression in H1299 cells (figure 4*b*,*c*). Other than DNMT1, 2-HG was reported to inhibit the TET family proteins that convert 5mC to 5-hydroxylmethylcytosine (5 hmC), resulting in an accumulation of 5 mC and thereby potentially altering the expression levels of large numbers of genes [26]. However, depletion of TET2 by siRNA had no effect on Fibulin-5 expression with or without 2-HG treatment in H1299 cells (figure 4*d*), suggesting TET2 is not involved in the methylation of Fibulin-5 promoter. DNMT1 inhibition by a suppressor repressed Fibulin-5 expression, which was inhibited by mutant IDH1 overexpressed in H1299 cells (figure 4*e*,*f*). The results indicate that IDH1 mutation in LC promotes the hypermethylation of Fibulin-5 promoter and represses Fibulin-5 expression.

### 3.5. Re-expression of Fibulin-5 inhibits migration and proliferation of non-small-cell lung cancer cells induced by IDH1 mutation

**Figure 5.**
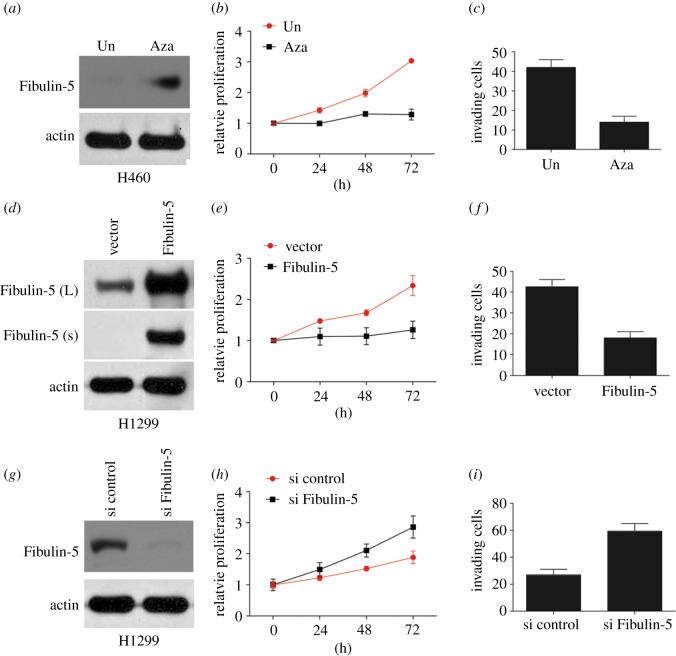
Fibulin-5 expression inhibits the effect of IDH1 mutation on NSCLC cells. (*a*) WB analysis was used to assess the expression of Fibulin-5 in H460 cells treated with 2 µM 5-aza for 4 days. (*b*) Proliferation of H460 cells treated as in (*a*) was assessed by MTS assay. (*c*) Migration of H460 cells treated as in (*a*) was assessed by Transwell migration assay. (*d*) WB analysis of Fibulin-5 expression in H1299 cells harbouring IDH1 mutation and transfected with the Fibulin-5 plasmid. (*e*) Proliferation of H1299 cells treated as in (*d*) was assessed by MTS assay. (*f*) Migration of H1299 cells treated as in (*d*) was assessed by Transwell migration assay. (*g*) WB analysis of Fibulin-5 expression in WT H1299 cells transfected with Fibulin-5 siRNA. (*h*) Proliferation of H1299 cells treated as in (*g*) was assessed by MTS assay. (*i*) Migration of H1299 cells treated as in (*g*) was assessed by Transwell migration assay.

To investigate whether IDH1 enhances the migration and proliferation of NSCLC cells through Fibulin-5, we examined the influence of Fibulin-5 expression in NSCLC cells. Consistent with a previous study [17], 5-Aza incubation recovered the expression of Fibulin-5 in H460 cells (figure 5*a*), and suppressed cell proliferation and migration (figure 5*b*,*c*). Transfection with Fibulin-5 plasmid in H1299 cells preliminarily supplemented with 2-HG or H460 cells also decreased cell proliferation and migration that were enhanced by 2-HG (figure 5*d*–*f*, figure 1*a*–*c*). By contrast, Fibulin-5 silencing by siRNA in H1299 cells enhanced proliferation and migration (figure 5*g*–*i*), similarly to the effects of 2-HG supplementation or transfection with IDH1 R132H. Furthermore, knockdown of Fibulin-5 in H460 cells also abolished the effects of IDH1 inhibitor, AGI, on the cell proliferation and migration (electronic supplementary material, figure S1D–F). These findings suggest that mutation in *IDH1* enhances proliferation and migration of NSCLC cells through inhibition of Fibulin-5 expression.

### 3.6. Fibulin-5 expression is reversely linked to IDH1 mutation in patients with non-small-cell lung cancer

**Figure 6.**
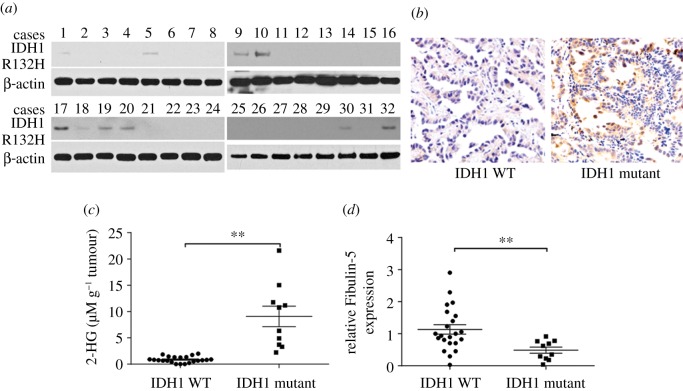
Fibulin-5 expression is reversely linked with IDH1 mutation in patients with NSCLC. (*a*) WB analysis of IDH1 R132H in malignant specimens from 40 patients with NSCLC. (*b*) Representative IHC staining for IDH1 R132H in IDH1 WT and mutant IDH1 specimens. (*c*) 2-HG levels in malignant specimens from the 40 patients with NSCLC. (*d*) Fibulin-5 mRNA levels in malignant specimens from the 40 patients with NSCLC.

To further explore the relationship between mutant IDH1 and Fibulin-5, we evaluated their expression in 40 patients with NSCLC. WB analysis and immunohistochemical (IHC) staining of IDH1 R132H were carried out. Ten patients with NSCLC were positive for IDH1 R132H mutation (figure 6*a*, *b*). We consequently categorized the patients into IDH1 WT and mutation groups, and evaluated 2-HG compound level and Fibulin-5 mRNA level. We discovered that 10 patients with the IDH1 mutation had elevated 2-HG levels in malignant tissues (figure 6*c*), in agreement with our findings *in vitro*. Nevertheless, Fibulin-5 mRNA levels in the 10 patients with NSCLC and IDH1 mutation were decreased compared with those in patients with WT IDH1 (figure 6*d*). Our results indicate that Fibulin-5 expression in patients with NSCLC is reversely linked with R132H mutation in *IDH1*.

## 4. Discussion

As well as malignancies of the central nervous system and leukaemia, mutations of IDH1 have been discovered in other solid cancers with low incidence [22]. A previous study has revealed that IDH1 transcription and translation are enhanced in NSCLC tissues compared with paired normal tissues and that IDH1 level may be a promising marker for NSCLC diagnosis [5]. Nevertheless, few studies have explored the impact of IDH1 mutation on NSCLC. Recent results on the activity of IDH1 mutation in cancer are contradictory. A study showed that IDH1 is a driver of cell immortalization and transformation, as well as generation of malignancies. Another study has reported that IDH1 mutation is related to reduced cell proliferation *in vitro* but favourable survival *in vivo* [27]. We show here that H1299 cells transfected with IDH1 R132H mutant displayed noticeably enhanced proliferation and migration compared with those transfected with IDH1 WT. We reveal that IDH1 R132H mutation enhanced proliferation and migration of NSCLC through 2-HG secretion, which consequently reduces Fibulin-5 expression.

Somatic IDH1 mutations were primarily identified in low-grade gliomas [12]. It has been shown using untargeted metabolic profiling by liquid chromatography-mass spectrometry that 2-HG expression increases by more than 100-fold in U87-MG cells that express the IDH1 mutation compared with cells that express WT IDH1 [12]. 2-HG binds to the α-KG-binding sites of the enzymes, TET2, and inhibits their function [10]. Aside from TETs, 2-HG also directly binds to DNMT1 and promotes its binding capability to the promoter of receptor-interacting protein 3 (RIP3) [25]. Generally, it was thought that IDH1 mutation leads to secretion of 2-HG, which leads to inhibition of TET2 or activation of DNMT1, and results in hypermethylation of large amount of genes [26]. The dysfunction of gene expression might therefore lead to cancer migration and proliferation. However, the excise methylation target of IDH1 mutation remains unclear. Here, we infer that secretion of 2-HG by IDH1 trigger NSCLC proliferation and migration by binding to DNMT1 can trigger a conformational alteration of the DNMT1 protein, which promotes its binding to the promoter of Fibulin-5 and suppressed its expression. However, further efforts are required to uncover the precise mechanism underlying 2-HG influence on DNMT1 activity.

DNA methylation plays important roles as epigenetic modulation that regulates gene expression. In mammalian cells, DNA methylation is carried out by three DNMTs: DNMT1, DNMT3b and DNMT3a [28]. DNMT1 is the most abundant DNMT in mammals, and is crucial in routine methylation pattern preservation throughout development and during the whole lifespan, whereas DNMT3a and DNMT3b is a de novo DNMT that constructs patterns of DNA methylation during the initial stage of development [29]. We show in our study that DNMT1 binding to the Fibulin-5 promoter relies on 2-HG, suggesting that DNMT1 modulation is a previously undiscovered activity of 2-HG. Further exploration of 2-HG binding site in DNMT1 is required.

Fibulin-5 is an inhibitor of LC invasion, and the epigenetic deactivation of Fibulin-5 participates in the progression of LC [16,17,30]. The downregulation of Fibulin-5 expression is prevalent in over 50% of LC, partly owing to the hypermethylation of promoters. Reduced expression of Fibulin-5 is related to a limited survival of patients with LC, as well as the progression of disease. Malignancy counteraction by Fibulin-5 is modulated in the malignant microenvironment through regulation of MMP-7, which is highly expressed in NSCLC and related to the limited clinical outcome [21]. We report in this study Fibulin-5 downregulation in LC, in agreement with previous research. Expression of Fibulin-5 is mainly reduced in LC through the hypermethylation of promoters. Hypermethylation of Fibulin-5 promoter has been identified in several histologically normal tissues surrounding the tumours. Nevertheless, the mechanism of methylation of Fibulin-5 promoters is still unclear. We demonstrate that Fibulin-5 silencing in NSCLC is linked to IDH1 mutation and that 2-HG enhances DNMT1 binding to the promoter of Fibulin-5, leading to its methylation.

## 5. Conclusion

Our results show an essential influence of IDH1 mutation on the inhibition of Fibulin-5 expression, as well as on the enhancement of migration and proliferation of NSCLC cells. Further studies that throw light upon the regulation of Fibulin-5 expression may offer innovative strategies to develop potential pharmacological or biological agents for the treatment of NSCLC with IDH1 mutation.

## Supplementary Material

Figure S1

## Data accessibility

This article has no additional data.
