# Supplementary figures and images for: IDH1 mutation promotes lung cancer cell proliferation through methylation of Fibulin-5
Source: Open Biol. 2018 Oct 10;8(10):180086. doi: 10.1098/rsob.180086 (PMC6223204; doi:10.1098/rsob.180086)

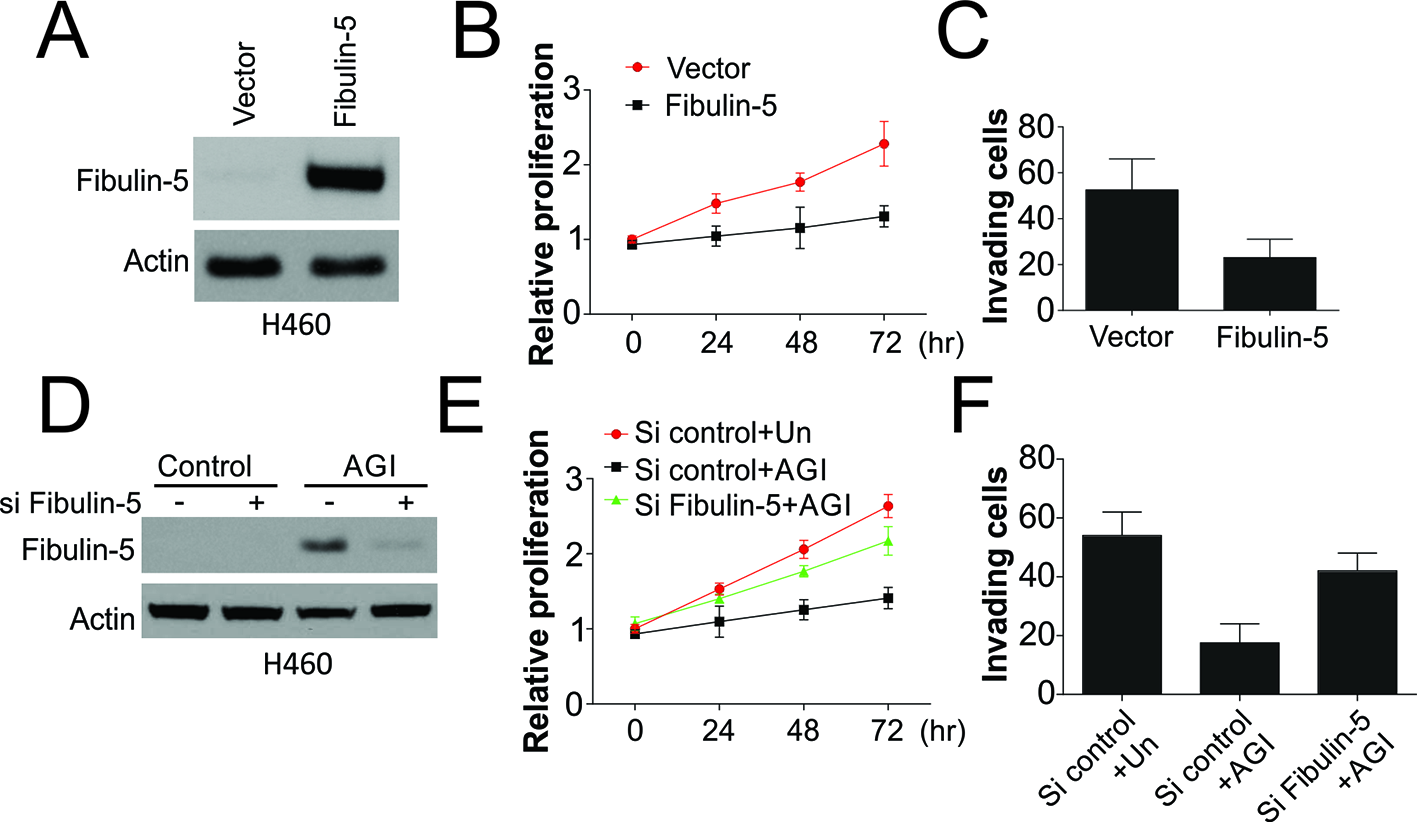

Supplement: Figure S1 [file rsob180086supp1.tif]
